# Gly-β-MCA is a potent anti-cholestasis agent against “human-like” hydrophobic bile acid-induced biliary injury in mice

**DOI:** 10.1016/j.jlr.2024.100649

**Published:** 2024-09-19

**Authors:** Mohammad Nazmul Hasan, Huaiwen Wang, Wenyi Luo, Yung Dai Clayton, Lijie Gu, Yanhong Du, Sirish K. Palle, Jianglei Chen, Tiangang Li

**Affiliations:** 1 Department of Biochemistry and Physiology, Harold Hamm Diabetes Center, University of Oklahoma Health Sciences Center, Oklahoma City, OK, USA; 2 Laboratory for Molecular Biology and Cytometry Research, University of Oklahoma Health Sciences Center, Oklahoma City, OK, USA; 3 Department of Pathology, Yale University, New Haven, CT, USA; 4 Department of Pediatrics, University of Oklahoma Health Sciences Center, Oklahoma City, OK, USA

**Keywords:** liver, bile acid, CYP7A1, nuclear receptor, drug therapy, FXR, cholangiopathy

## Abstract

Cholestasis is a chronic liver disease with limited therapeutic options. Hydrophobic bile acid-induced hepatobiliary injury is a major pathological driver of cholestasis progression. This study investigates the anti-cholestasis efficacy and mechanisms of action of glycine-conjugated β-muricholic acid (Gly-β-MCA). We use female *Cyp2c70* KO mice, a rodent cholestasis model that does not produce endogenous muricholic acid (MCA) and exhibits a “human-like” hydrophobic bile acid pool and female-dominant progressive hepatobiliary injury and portal fibrosis. The efficacy of Gly-β-MCA and ursodeoxycholic acid (UDCA), the first line drug for cholestasis, on cholangiopathy and portal fibrosis are compared. At a clinically relevant dose, Gly-β-MCA shows comparable efficacy as UDCA in reducing serum transaminase, portal inflammation and ductular reaction, and better efficacy than UDCA against portal fibrosis. Unlike endogenous bile acids, orally administered Gly-β-MCA is absorbed at low efficiency in the gut and enters the enterohepatic circulation mainly after microbiome-mediated deconjugation, which leads to taurine-conjugated MCA enrichment in bile that alters enterohepatic bile acid pool composition and reduces bile acid pool hydrophobicity. Gly-β-MCA also promotes fecal excretion of endogenous hydrophobic bile acids and decreases total bile acid pool size, while UDCA treatment does not alter total bile acid pool. Furthermore, Gly-β-MCA treatment leads to gut unconjugated MCA enrichment and reduces gut hydrophobic lithocholic acid (LCA) exposure. In contrast, UDCA treatment drives a marked increase of LCA flux through the large intestine. In conclusion, Gly-β-MCA is a potent anti-cholestasis agent with potential clinical application in treating human cholestasis.

Cholestasis is a chronic liver disease due to impaired bile flow out of the liver, causing hepatic accumulation of bile acids that damage the liver parenchyma and biliary tree (1). Chronic cholestasis causes liver fibrosis and increases the risk of developing end stage liver diseases and liver and bile duct cancer. Ursodeoxycholic acid (UDCA, Ursodiol™), a hydrophilic and non-cytotoxic primary bile acid found abundantly in bears but not synthesized in significant amount in humans or mice, has been the only first line treatment for primary biliary cholangitis (PBC) since its FDA approval in 1997. UDCA decreases the hydrophobicity and cytotoxicity of biliary bile acid pool, promotes biliary bile acid secretion, and shows anti-apoptosis effect (2). However, clinical data show that up to 40% of patients with PBC do not adequately respond to UDCA treatment, leaving these patients with limited treatment options (3, 4). Obeticholic acid (OCA, Ocaliva) is a potent farnesoid x receptor (FXR) agonist approved by the FDA in 2016 as a second line drug for patients with PBC who do not respond adequately to or cannot tolerate UDCA treatment (5, 6, 7, 8). OCA treatment is commonly associated with pruritus, which is the leading cause of treatment discontinuation among patients with PBC. Unfortunately, most other forms of genetic and acquired cholestasis still do not have an effective pharmacological treatment, highlighting the unmet need for developing additional safe and effective anti-cholestasis therapeutics.

Regardless of etiology, hepatobiliary bile acid toxicity is a shared major pathological driver of cholestasis progression. Chenodeoxycholic acid (CDCA) and cholic acid (CA) are the two primary bile acids synthesized from cholesterol in human livers (9). In small and large intestines, bacterial enzymes modify primary bile acids to convert CDCA and CA to secondary bile acids lithocholic acid (LCA) and deoxycholic acid (DCA), respectively. In mice, most of the CDCA is converted to muricholic acid (MCA) in the liver by the mouse-specific CYP2C70 enzyme (10, 11), resulting in CA and MCA being the major primary bile acids in mice. Compared to the hydrophobic CDCA, MCA is highly hydrophilic and non-cytotoxic. Genetic deletion of the *Cyp2c70* gene in mice abolished MCA synthesis, rendering *Cyp2c70* KO mice a “human-like” bile acid pool consisting of taurine-conjugated CDCA and CA as the primary bile acids (10, 11). However, due to exposure to a bile acid pool with increased hydrophobicity, *Cyp2c70* KO mice developed spontaneous biliary injury and portal fibrosis, which were more severe in females than males. In the past few years, *Cyp2c70* KO mice have been increasingly adopted as a valuable rodent cholestasis model for the study of “human-like” bile acid pool-induced hepatobiliary injury (12, 13, 14).

While CDCA is a potent FXR ligand, MCAs act as endogenous FXR antagonists (15). Remodeling of the gut microbiome that increased MCA abundance reduced intestine FXR activity and improved metabolic homeostasis (16). In human livers, bile acids are conjugated to either glycine or taurine, while bile acids in mice are almost exclusively conjugated to taurine. Molecular modeling subsequently identified glycine-conjugated β-MCA (Gly-β-MCA), a hydrophilic bile acid that is not abundantly synthesized in humans or mice, as an FXR antagonist with anti-obesity and insulin-sensitizing effects in obese mice (17). It was previously shown that Gly-β-MCA was not absorbed in the small intestine and was highly resistant to bacterial bile salt hydrolase (BSH)-mediated deconjugation, and therefore acted in a “gut-restricted manner” to inhibit intestine FXR (17).

Taking advantage of the lack of endogenous MCAs in *Cyp2c70* KO mice, we recently revealed that Gly-β-MCA exhibited anti-cholestasis and anti-fibrosis effects (18). However, our prior study only tested the effect of Gly-β-MCA in male *Cyp2c70* KO mice that exhibited mild hepatobiliary injury. In these mice, only relatively modest improvement of liver pathology was observed at a Gly-β-MCA dose of 20 mg/kg/day, which translated into a human equivalent dose of ∼1.6 mg/kg/day that was significantly lower than the optimal UDCA dose of 13–15 mg/kg/day used to treat patients with PBC. These limitations of our prior study left the anti-cholestasis efficacy and mechanisms-of-action of Gly-β-MCA incompletely characterized. Therefore, the goal of this follow-up study is to further elucidate the therapeutic effects and mechanisms of Gly-β-MCA in reference to the first line anti-cholestasis drug UDCA at a clinically relevant dose in female *Cyp2c70* KO mice that developed progressive and severe hepatobiliary injury and fibrosis. This study showed that orally administered Gly-β-MCA followed a unique gut microbiome-dependent enterohepatic circulation to modulate the endogenous bile acid metabolism. At the same dose, Gly-β-MCA was equally effective as UDCA in reducing ductular reaction and portal inflammation, and more effective than UDCA in reducing liver fibrosis. These findings suggest that Gly-β-MCA is a potent anti-cholestasis agent with potential clinical application in treating human cholestasis.

## Materials and Methods

### Reagent

Alanine aminotransferase (ALT) assay kit, alkaline phosphatase (ALP) assay kit, and total bilirubin assay kit were purchased from Pointe Scientific. BR2 bilirubin calibrator was purchased from Verichem Laboratories Inc. Gly-β-MCA was purchased from MedChemExpress LLC. Bile acid assay kit was purchased from Diazyme Laboratories. F4/80 antibody (Cat #.70076) was purchased from Cell Signaling Technology. CK19 antibody (ab52625) was purchased from Abcam. Chenodeoxycholic acid-d4 MaxSpec® standard (CDCA-d4, No. 31366), taurochenodeoxycholic acid-d4 MaxSpec® standard, (T-CDCA-d4, No. 31362), ursodeoxycholic acid-d4 MaxSpec® standard (UDCA-d4, No. 31368) and tauroursodeoxycholic acid-d4 MaxSpec® standard (T-UDCA-d4, No. 31564), and lithocholic acid-d4 MaxSpec® standard (LCA-d4, No. 31354) were purchased from Caymen Chemical Company. UDCA (U5127) was purchased from Sigma Aldrich.

### Mice and treatments

The *Cyp2c70* KO mice on C57BL/6J genetic background with Exon 4 deletion were generated by CRISPR/Cas-mediated genome engineering as described previously (14). WT mice were obtained by breeding *Cyp2c70* ± mice. The *Cyp2c70* KO mice were obtained by breeding *Cyp2c70* KO mice. It was reported previously that cholestasis in *Cyp2c70* KO mice was more severe in females than males (12, 14). In this study, we only used female *Cyp2c70* KO mice with more severe liver pathology to compare the therapeutic efficacy and mechanisms of Gly-β-MCA and UDCA against cholestasis liver injury. Male *Cyp2c70* KO mice were not used in this study because we have previously studied the protective effect of Gly-β-MCA in male *Cyp2c70* KO mice (18). Mice were housed in micro-isolator cages under 7 am to 7 pm light cycle and 7 pm -7 am dark cycle. Gly-β-MCA or UDCA was mixed with chow diet to achieve an estimated 160 mg/kg/day intake based on 4 g/day food intake by a 25 g mouse. These treatments were initiated when mice were 8 weeks old for 4 weeks. Mice were then euthanized at 12 weeks of age following a 6-h fast from 9 am to 3 pm and tissues and blood samples were collected. To study Gly-β-MCA metabolism in vivo, *Cyp2c70* KO mice were orally gavaged with 0.5 mg Gly-β-MCA (in 0.5% methylcellulose in 200 μl volume) and fasted for 1, 4 or 16 h before tissue collection. Animals received humane care according to the criteria outlined in the “Guide for the Care and Use of Laboratory Animals.” All animal studies were approved by the Institutional Animal Care and Use Committee of the University of Oklahoma Health Sciences Center (Approval # 22-072-EAFHI).

### Liver pathology

Liver fibrosis, inflammation, and bile ductular reaction/proliferation were evaluated by a clinical liver pathologist in a blinded fashion. Portal and periportal inflammation were scored as previously described (19): score 0 if no inflammation or minimal inflammation in rare portal tracts is present; score 1 if inflammation is present in more than rare but less than one-third of portal tracts; score 2 if inflammation is present in equal or greater than one third to two-thirds of portal tracts; score 3 if inflammation is present in greater than two-thirds of portal tracts. Lobular inflammation was scored as follows: score 0 if there is no lobular inflammation or less than 1 inflammatory focus per 20× field; score 1 if there are equal or less than two inflammatory foci per 20× field; score 2 if there are 2–4 inflammatory foci per 20× field; score 3 if there are greater than 4 inflammatory foci per 20× field. The scoring of bile ductular reaction/proliferation was adapted as previously described (20): score 0 if there are less than 5 bile ducts per portal tract, score 1 if there are 5–9 bile ducts per portal tract; score 2 if there are equal or greater than 10 bile ducts per portal tract; score 3 if there are equal or greater than 10 bile ducts per portal tract with prominent lobular extension. Fibrosis was scored as follow: score 0 if there is no fibrosis; score 1 if there is portal fibrous expansion with or without periportal fibrosis involving zone 1; score 2 if there is portal fibrous expansion with periportal fibrosis extending beyond zone 1.

### Bile acid analysis

This analysis was performed as described in detail previously (14). Briefly, bile acids were extracted from the liver (∼50 mg), whole gallbladder bile, whole small intestine with content, and dried feces in 95% ethanol at 50°C overnight. The extracts were centrifuged at 10,000×*g* for 10 min at room temperature and an aliquot of the supernatant was used to measure total bile acids with an assay kit. Total liver bile acid content was calculated based on the whole liver weight. The bile acid pool was calculated as the sum of total bile acids in the whole liver, gallbladder, and small intestine. To collect fecal samples, an individual mouse was placed in a jar, and fresh feces were collected. LC-MS method was used to measure individual bile acid species in various bile acid extracts as previously described (14). The bile acid pool hydrophobicity index was calculated according to a previous report (19). The hydrophobicity index of α-MCA, β-MCA, and LCA were not available, and the hydrophobicity index values of their taurine conjugates were used in the calculation.

### Real-time PCR

Total tissue RNA was purified with Trizol reagent (Sigma-Aldrich). Reverse transcription was performed by using SuperScript III reverse transcriptase and Oligo dT primer (ThermoFisher Scientific). Real-time PCR was performed on a Bio-Rad CFX384 Real-time PCR system with iQ SYBR Green Supermix (Bio-rad). The comparative CT (Ct) method was used to determine the relative mRNA expression with 18S used for normalization. The control group was arbitrarily set as “1”.

### In vitro bile acid deconjugation measurement

The in vitro reaction was carried out under similar conditions as previously reported (14, 17). Fresh fecal samples were collected from *Cyp2c70* KO mice by placing individual mice in a jar. Fresh fecal samples were resuspended in reaction buffer (10% PBS and 90% 3 mM sodium acetate, pH = 5.2) to a final suspension of 4 mg feces/ml. This suspension was evenly divided into Eppendorf tubes (0.5 ml/tube) and T-CDCA-d4, T-UDCA-d4 or Gly-β-MCA was added to a final concentration of 40 μM and incubated at 37°C for 6 h. Methanol (0.5 ml) was added to each tube followed by incubation on ice for 30 min to precipitate proteins. After centrifugation at 14,000 × g for 10 min, supernatant was vacuum dried and resuspended in sample buffer for LC-MS analysis. Gly-β-MCA, β-MCA, T-CDCA-d4, CDCA-d4, T-UDCA-d4, UDCA-d4, and LCA-d4 were measured by LC-MS. LCA-d4 was not detectable in all samples, suggesting that CDCA-d4 or UDCA-d4 was not converted to LCA-d4 in measurable amounts in vitro. Therefore, the deconjugation rate of T-CDCA-d4 was estimated as CDCA-d4/(T-CDCA-d4+CCA-d4) and the deconjugation rate of T-UDCA-d4 was estimated as UDCA-d4/(T-UDCA-d4+UDCA-d4). The deconjugated rate of Gly-β-MCA was estimated as β-MCA/(Gly-β-MCA+ β-MCA).

### Statistical analysis

All results were expressed as mean ± SEM. GraphPad Prism 10 was used for statistical analysis (one-way ANOVA or Student’s *t* test). A *P* < 0.05 was considered statistically significant. “∗”, <0.05; “∗∗”, <0.01; “∗∗∗”, <0.001; and “∗∗∗∗” <0.0001.

## Results

### Gly-β-MCA treatment is equally effective as UDCA in alleviating ductular reaction and portal inflammation in female *Cyp2c70 KO* mice

**Fig. 1:**
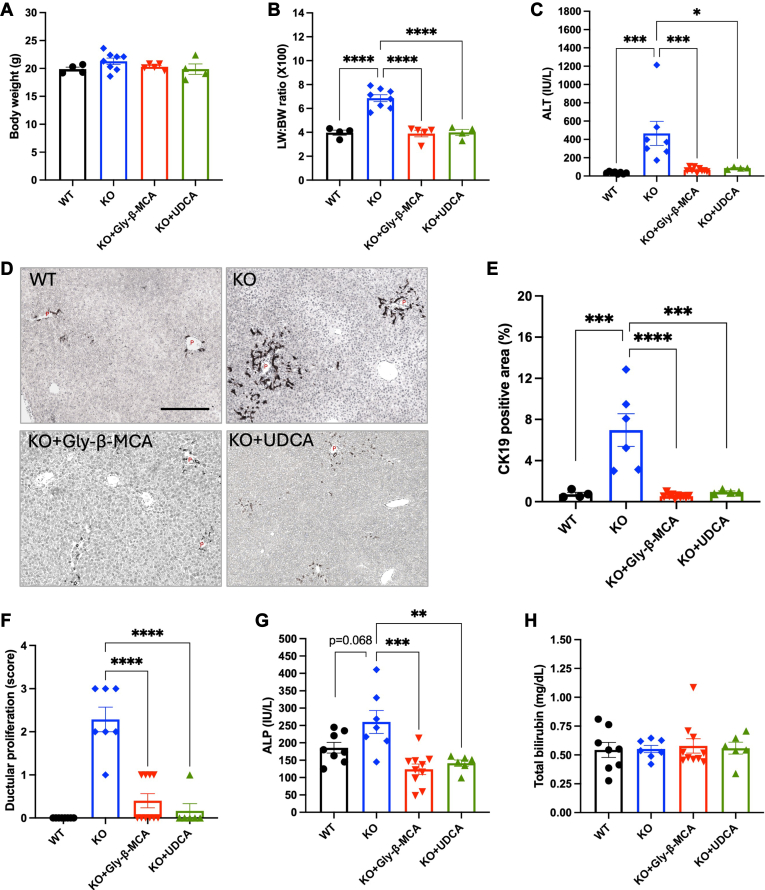
Gly-β-MCA and UDCA treatment reduce liver injury and ductular reaction in female *Cyp2c70* KO mice. Female WT and *Cyp2c70* KO mice at 8 weeks of age were treated with Gly-β-MCA or UDCA (160 mg/kg/day) for 4 weeks. Mice were fasted for 6 h from 9 am-3 pm and euthanized. A: Body weight after treatment. B: Liver weight (LW): body weight (BW) ratio. C: Serum ALT. D: Representative images of immunohistochemistry of CK19 stain. Scale bar = 250 um. P: portal vein. E: CK19 positive area per view was quantified with ImageJ. Software. F: Histology score of ductular proliferation. G: Serum ALP. H: Serum total bilirubin. n = 4–10. All results are expressed as mean ± SEM. One-way ANOVA and Tukey post hoc test were used for all statistical analysis. A *P* < 0.05 was considered statistically significant. “∗”, <0.05; “∗∗∗”, <0.001; “∗∗∗∗” <0.0001.

**Fig. 2:**
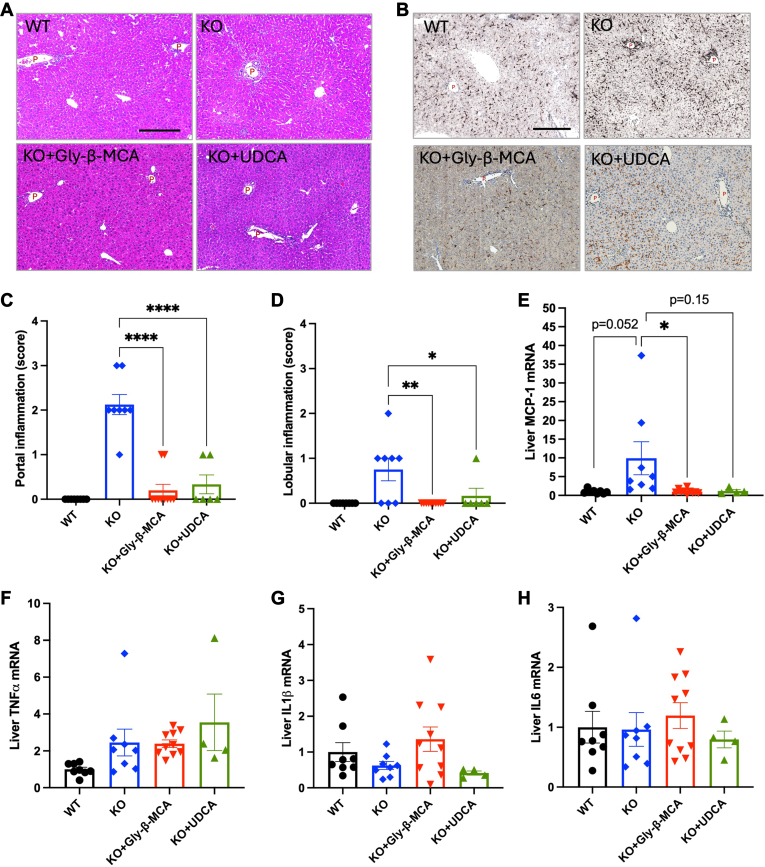
Gly-β-MCA and UDCA treatment reduce portal inflammatory infiltration in female *Cyp2c70* KO mice. Female WT and *Cyp2c70* KO mice at 8 weeks of age were treated with Gly-β-MCA or UDCA (160 mg/kg/day) for 4 weeks. Mice were fasted for 6 h from 9 am-3 pm and euthanized. A: Representative images of liver H&E stain. Scale bar = 250 um. P: portal vein. B: Representative images of immunohistochemistry of F4/80 stain. Scale bar = 250 um. P: portal vein. C: Pathology score of portal inflammation. D: Pathology score of lobular inflammation. E, F, G, H: Liver mRNA expression. n = 4–10. All results are expressed as mean ± SEM. One-way ANOVA and Tukey post hoc test were used for all statistical analysis. A *P* < 0.05 was considered statistically significant. “∗”, <0.05; “∗∗”, <0.01; “∗∗∗∗” <0.0001.

To determine the anti-cholestasis efficacy of Gly-β-MCA in comparison to that of UDCA, we treated female *Cyp2c70* KO mice with either Gly-β-MCA or UDCA at a dose of ∼160 mg/kg/day (based on 16 g/100 g BW daily food intake) for 4 weeks. This dose used in mice translates into the human equivalent dose of ∼13 mg/kg/day, which is the optimal dose of UDCA (13–15 mg/kg/day) used to treat patients with PBC (21) and has previously been shown to alleviate hepatobiliary injury and fibrosis in female *Cyp2c70* KO mice (12). Compared to WT mice, the *Cyp2c70* KO mice showed hepatomegaly (Fig 1A, B) and elevated serum transaminase level (Fig. 1C), which was consistent with hepatobiliary injury. Both Gly-β-MCA and UDCA treatment fully normalized liver size and serum transaminase to the level of WT mice (Fig. 1A–C). Immunohistochemistry (IHC) staining of cytokeratin 19 (CK19) and liver pathology analysis of ductular proliferation confirmed that *Cyp2c70* KO mice developed extensive ductular reaction, which was significantly prevented by either Gly-β-MCA or UDCA treatment (Fig. 1D–F). Consistently, serum ALP of *Cyp2c70* KO mice was elevated compared to WT mice (*P* = 0.068) and was significantly decreased by Gly-β-MCA or UDCA treatment (Fig. 1G). Serum total bilirubin was not significantly increased in the *Cyp2c70* KO mice compared to WT mice (Fig. 1H), suggesting that bile flow was not likely severely obstructed in the *Cyp2c70* KO mice despite biliary injury. H&E staining and IHC staining of macrophage marker F4/80 revealed that *Cyp2c70* KO mice showed portal inflammatory infiltration, which was also fully prevented by either Gly-β-MCA or UDCA treatment (Fig 2A, B). Histological analysis revealed that portal inflammation (scored at 2 or 3) was present in most of the *Cyp2c70* KO mice and was mostly reversed by either Gly-β-MCA or UDCA treatment (Fig. 2C). In contrast, only mild or no lobular inflammation (scored 0 or 1) was noted in most of the *Cyp2c70* KO mice, and almost all *Cyp2c70* KO mice treated with either Gly-β-MCA or UDCA treatment showed no lobular inflammation (Fig. 2D). Interface hepatitis and cholangitis were very rarely present in the *Cyp2c70* KO mice. Measurement of hepatic mRNA expression showed that although chemokine Monocyte Chemoattractant Protein-1 (MCP-1) mRNA was induced in *Cyp2c70* KO mice compared to WT mice and was reduced upon Gly-β-MCA or UDCA treatment, the mRNA of hepatic inflammatory cytokines tumor necrosis factor α (TNFα), interleukin 1β (IL1β) and interleukin 6 (IL6) showed no significant induction in *Cyp2c70* KO mice compared to WT mice and was not altered by Gly-β-MCA or UDCA treatment (Fig. 2E–H). The lack of correlative changes of total hepatic cytokine mRNA expression was unclear but could be because inflammatory infiltration in *Cyp2c70* KO mice was mainly confined to the portal areas without profound parenchymal inflammatory infiltration (Fig 2C, D). In summary, these results showed that Gly-β-MCA was equally effective as UDCA in reducing hepatobiliary injury, ductular reaction, and portal inflammation in female *Cyp2c70* KO mice.

### Gly-β-MCA was more effective than UDCA in reducing portal fibrosis in female *Cyp2c70* KO mice

**Fig. 3:**
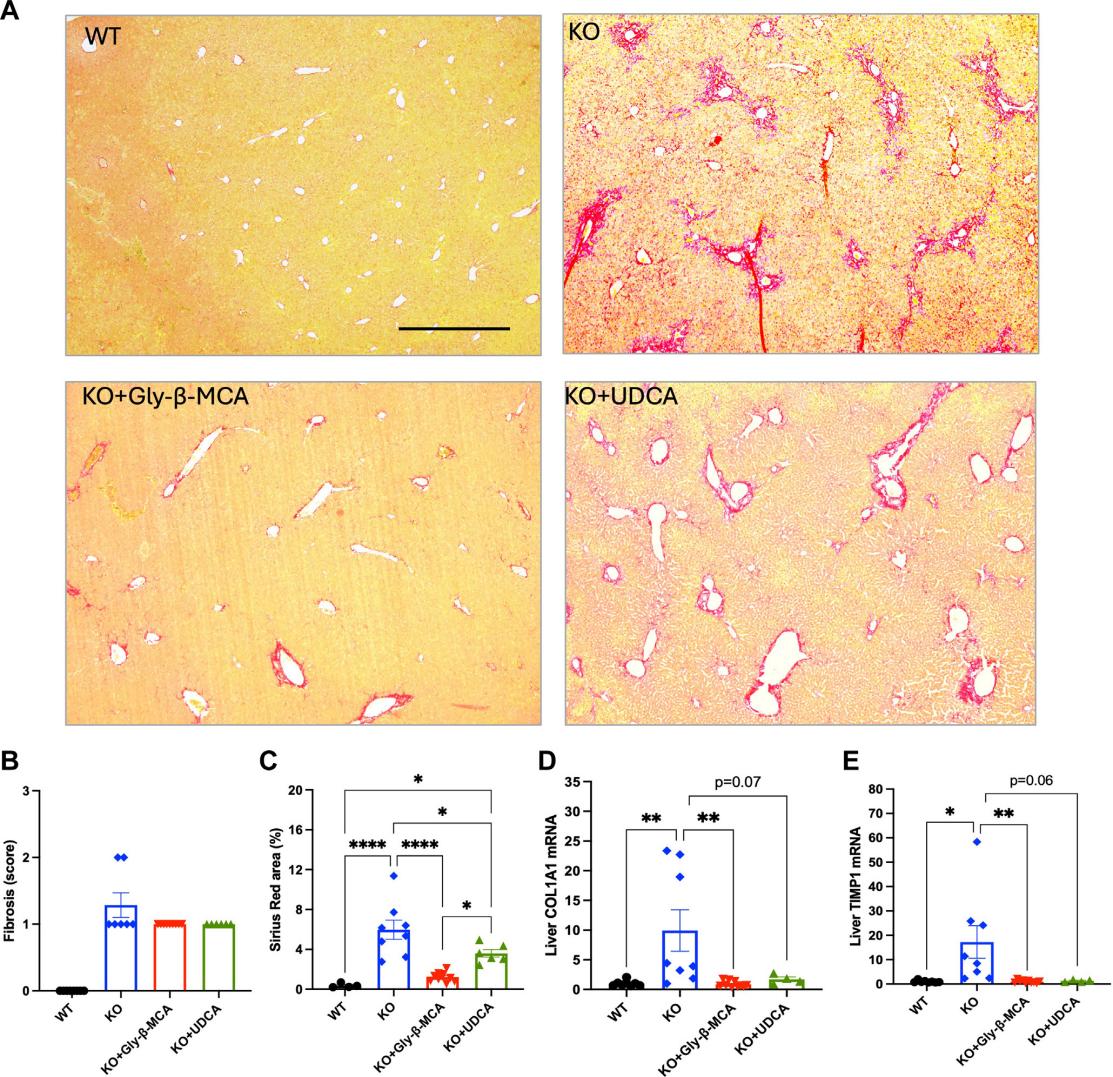
Gly-β-MCA and UDCA treatment reduce portal fibrosis in female *Cyp2c70* KO mice. Female WT and *Cyp2c70* KO mice at 8 weeks of age were treated with Gly-β-MCA or UDCA (160 mg/kg/day) for 4 weeks. Mice were fasted for 6 h from 9 am-3 pm and euthanized. A: Representative images of Sirius Res stain. Scale bar = 600 um. B: Liver fibrosis score. C: Sirius Red positive area per view was quantified with ImageJ. Software. D, E: Liver mRNA expression. n = 4–10. All results are expressed as mean ± SEM. One-way ANOVA and Tukey post hoc test were used for all statistical analysis. A *P* < 0.05 was considered statistically significant. “∗”, <0.05; “∗∗”, <0.01; “∗∗∗∗” <0.0001.

Liver Sirius Red stain revealed significant portal fibrosis in the *Cyp2c70* KO mice and both Gly-β-MCA and UDCA treatment attenuated portal fibrosis in the *Cyp2c70* KO mice (Fig. 3A). Histology analysis revealed that liver fibrosis in the *Cyp2c70* KO mice was mainly confined to the periportal area (Score 1) with less than half of the *Cyp2c70* KO mice showing fibrosis extending beyond zone 1 (Score 2) (Fig. 3B). Upon Gly-β-MCA or UDCA treatment, all mice showed periportal fibrosis without extension beyond zone 1 (score 1) (Fig. 3B). Because the histological scoring method only considered the qualitative distribution of fibrosis in the liver, we next performed quantitative measurement of Sirius Red positive area and found that Gly-β-MCA treatment was significantly more effective than UDCA in attenuating the degree of portal fibrosis in the *Cyp2c70* KO mice (Fig. 3C). However, this different anti-fibrosis efficacy between Gly-β-MCA and UDCA was not reflected at the hepatic fibrosis gene expression level, with the induction of hepatic collagen, type I, α1 (COL1A1) and tissue inhibitor of metalloproteinase 1 (TIMP1) mRNA fully normalized to the level of WT mice (Fig 3D, E), which was consistent with reduced liver fibrosis in the treated mice (Fig. 3C).

### Gly-β-MCA, but not UDCA, reduced the total bile acid pool in female *Cyp2c70* KO mice

**Fig. 4:**
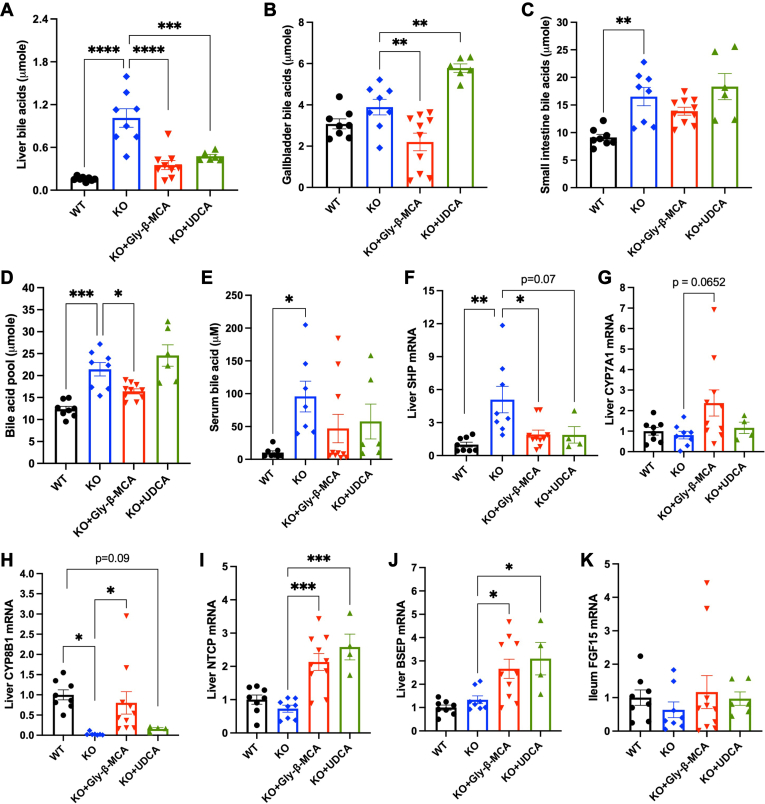
Gly-β-MCA and UDCA treatment effects on bile acid pool in female *Cyp2c70* KO mice. Female WT and *Cyp2c70* KO mice at 8 weeks of age were treated with Gly-β-MCA or UDCA (160 mg/kg/day) for 4 weeks. Mice were fasted for 6 h from 9 am-3 pm and euthanized. A, B, C, D: Tissue total bile acid content and total bile acid pool. E: Serum bile acid concentration. F, G, H, I, J: Liver mRNA expression. K: Ileum mRNA expression. n = 4–10. All results are expressed as mean ± SEM. One-way ANOVA and Tukey post hoc test were used for all statistical analysis. A *P* < 0.05 was considered statistically significant. “∗”, <0.05; “∗∗”, <0.01; “∗∗∗”, <0.001; “∗∗∗∗” <0.0001.

To better understand the mechanisms mediating the beneficial effects of Gly-β-MCA treatment, we next determined the effect of Gly-β-MCA and UDCA on bile acid metabolism. Compared to WT mice, *Cyp2c70* KO mice showed increased hepatic and intestine bile acids and a ∼2-fold enlarged bile acid pool (Figs. 4, *A*–*D*), which was consistent with our previous report (14). Hepatic bile acid accumulation in *Cyp2c70* KO mice was significantly reduced to similar levels by Gly-β-MCA and UDCA treatment (Fig. 4A). Gly-β-MCA also significantly decreased gallbladder bile acids but not small intestine total bile acids (Fig 4B, C), and significantly decreased total bile acid pool (Fig. 4D). In contrast, UDCA treatment increased gallbladder bile acids and did not reduce small intestine bile acids or total bile acid pool (Fig. 4B–D). *Cyp2c70* KO mice also showed significantly elevated serum bile acids compared to WT (Fig. 4E). Despite significantly alleviated hepatobiliary injury, the mean serum bile acid concentration was not significantly reduced by Gly-β-MCA or UDCA treatment. However, it was noted that serum bile acid concentration in ∼70% of the Gly-β-MCA-treated group (7 out of 10) and the UDCA-treated group (4 out of 6) returned to comparable level of the WT mice (Fig. 4E).

Consistent with hepatic bile acid levels, FXR target small heterodimer partner (SHP) mRNA increased in *Cyp2c70* KO mice compared to WT and was reduced by Gly-β-MCA and UDCA treatment compared to the *Cyp2c70* KO controls (Fig. 4F). The mRNA expression of the *Cyp7a1* gene, encoding the rate-limiting enzyme in bile acid synthesis cholesterol 7α-hydroxylase (CYP7A1), was induced by Gly-β-MCA treatment (*P* = 0.07) but was not affected by UDCA treatment (Fig. 4G). The mRNA expression of the *Cyp8b1* gene, encoding sterol 12α-hydroxylase (CYP8B1) enzyme that mediates CA synthesis, was markedly lower in *Cyp2c70* KO mice compared to WT mice (Fig. 4H). CYP8B1 mRNA was significantly induced upon Gly-β-MCA but not UDCA treatment (Fig. 4H). The mRNA of Na^+^-dependent taurocholate co-transporting polypeptide (NTCP), which mediates hepatocyte basolateral bile acid uptake, was induced by both Gly-β-MCA and UDCA treatment (Fig. 4I). CYP7A1, CYP8B1, and NTCP are known to be repressed by bile acids and FXR, and their induction may reflect lower hepatic bile acid concentration (9). Paradoxically, we found that Gly-β-MCA and UDCA treatment did not decrease but increased hepatic mRNA expression of bile salt export pump (BSEP), which is known to be positively regulated by FXR (Fig. 4J). Induction of BSEP and NTCP may facilitate trans-hepatic bile acid flux and biliary bile acid secretion. Neither Gly-β-MCA nor UDCA affected ileum FGF15 mRNA expression (Fig. 4K).

### Orally administered Gly-β-MCA enters the enterohepatic circulation to modulate bile acid pool composition

**Fig. 5:**
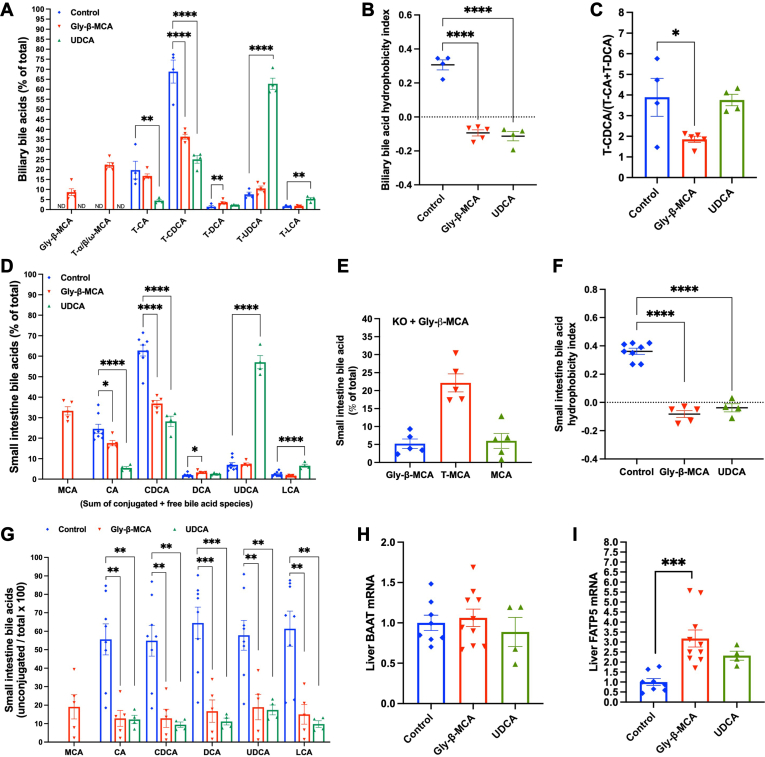
Gly-β-MCA and UDCA treatment effects on bile acid pool composition in female *Cyp2c70* KO mice. Female *Cyp2c70* KO mice at 8 weeks of age were treated with Gly-β-MCA or UDCA (160 mg/kg/day) for 4 weeks. Mice were fasted for 6 h from 9 am-3 pm and euthanized. A: Biliary bile acid composition. B: Biliary bile acid pool hydrophobicity index. C: Biliary T-CDCA: (T-CA + T-DCA) ratio. D: Small intestine bile acid composition. The values represent the sum of conjugated and unconjugated bile acid of each species. E: Small intestine MCA species of female *Cyp2c70* KO mice treated with Gly-β-MCA for 4 weeks. T-MCA is the sum of T-α/β/ω-MCAs. MCA is the sum of α/β-MCAs. F: Small intestine bile acid pool hydrophobicity index. G: Small intestine unconjugated bile acids (% of the sum of total conjugated and unconjugated bile acids of each species). H, I: Liver mRNA. n = 4–5 for A-G. n = 4–10 for H-I. All results are expressed as mean ± SEM. One-way ANOVA and Tukey post hoc test were used for all statistical analysis. A *P* < 0.05 was considered statistically significant. “∗”, <0.05; “∗∗”, <0.01; “∗∗∗”, <0.001; “∗∗∗∗” <0.0001.

Consistent with previous report (14), bile acids in the gallbladder bile of *Cyp2c70* KO mice consisted of predominantly taurine-conjugated (T-) CDCA (T-CDCA) (∼70%) and a relatively lower abundance of T-CA (∼20%) and T-UDCA (∼8%) (Fig. 5A). Interestingly, while *Cyp2c70* KO mice did not synthesize MCAs, Gly-β-MCA treatment caused Gly-β-MCA to be enriched to ∼10% and T-MCAs to be enriched to ∼20% in the bile (Fig. 5A). The ∼30% MCA enrichment diluted the biliary T-CDCA abundance and significantly lowered bile acid hydrophobicity index compared to that of the untreated *Cyp2c70* KO mice (Fig 5A, B). Gly-β-MCA treatment did not significantly reduce the relative abundance of T-CA possibly because of increased hepatic CYP8B1 expression and T-CA synthesis (Fig. 4H), which was supported by decreased T-CDCA to 12-hydroxylated bile acid (T-CA + T-DCA) ratio (Fig. 5C). As expected, UDCA treatment caused T-UDCA to be enriched to ∼65% in gallbladder bile and significantly lowered the bile acid hydrophobicity (Fig 5A, B). UDCA treatment did not alter CYP8B1 expression or T-CDCA to 12-hydroxylated bile acid (T-CA + T-DCA) ratio (Figs. 4H and 5C), which provided an explanation that UDCA lowered the relative abundance of both T-CA and T-CDCA while Gly-β-MCA treatment only lowered the relative abundance of T-CDCA (Fig. 5A).

Because bile is released into the small intestine, small intestine bile acid composition was very similar to the bile acid composition of the gallbladder bile, with conjugated and unconjugated CDCA (∼60%), CA (∼25%), and UDCA (∼8%) accounting for the majority of the small intestine bile acids of *Cyp2c70* KO mice (Fig. 5D). In small intestine of Gly-β-MCA-treated *Cyp2c70* KO mice, total MCA was enriched to ∼30% (Fig. 5D), which consisted of ∼5% Gly-β-MCA, ∼20% T-α/β/ω-MCAs, and ∼5% unconjugated α/β-MCA (Fig. 5E). The relative abundance of CDCA and CA was significantly decreased in Gly-β-MCA-treated mice (Fig. 5D). As a result, Gly-β-MCA also significantly decreased small intestine bile acid hydrophobicity index (Fig. 5F). UDCA treatment significantly increased UDCA abundance to ∼60%, decreased the relative abundance of CDCA and CA, and decreased bile acid hydrophobicity index in the small intestine (Fig 5D, F). We also found that significant deconjugation of bile acids already occurred in small intestine, with slightly more than 50% of all bile acid species found in the unconjugated form in *Cyp2c70* KO mice (Fig. 5G). In contrast, we found that only less than 20% of all bile acid species, including MCA and UDCA, was in the unconjugated form in mice treated with Gly-β-MCA or UDCA (Fig. 5G), suggesting reduced bile acid deconjugation in small intestine of these mice. Hepatic mRNA expression of the bile acid conjugation gene bile acid-CoA:amino acid N-acyltransferase (BAAT) was not altered by Gly-β-MCA or UDCA treatment (Fig. 5H). Gly-β-MCA and UDCA increased the mRNA expression of fatty acid transport protein 5 (FATP5), a bile acid CoA lyase mediating the first step in bile acid conjugation (22, 23), although the UDCA effect was not statistically significant (Fig. 5I). FATP5 mRNA induction by Gly-β-MCA and UDCA was possibly a result of reduced FXR repression of FATP5 (24). However, total unconjugated bile acids accounted for less than 1% of total gallbladder bile acids in all 3 groups of mice, suggesting that reduced unconjugated bile acids in small intestine of Gly-β-MCA or UDCA treated mice was unlikely resulted from altered hepatic bile acid conjugation. We speculate that increased total intestine bile acid flux because of exogenous Gly-β-MCA or UDCA administration may saturate the bacterial BSH activity due to relatively low abundance of microbiota in the small intestine. Other possible causes may include reduced abundance of BSH harboring bacteria due to an overall reduction of small intestine bacteria or altered small intestine microbiome composition upon Gly-β-MCA or UDCA treatment. Future studies are still needed to determine the effect of Gly-β-MCA or UDCA on gut microbiome in *Cyp2c70* mice.

**Fig. 6:**
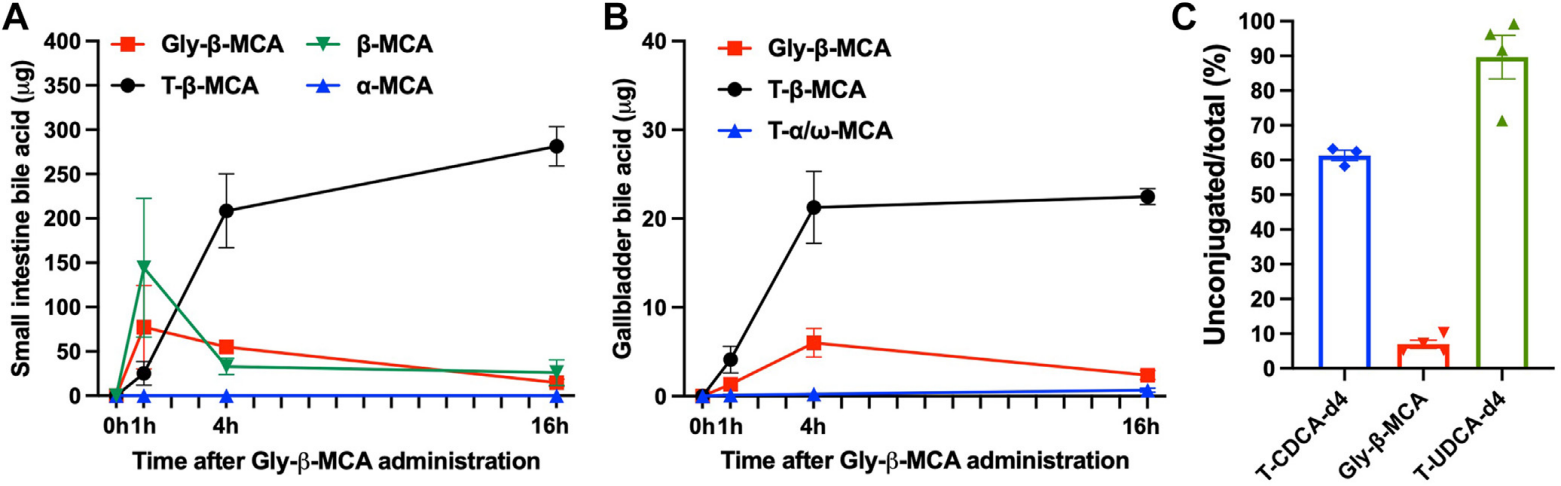
Gly-β-MCA metabolism in *Cyp2c70* KO mice. A single bolus of 0.5 mg Gly-β-MCA was administered to mice via oral gavage, and gallbladder and small intestine bile acids were analyzed by LC-MS. A: Small intestine bile acids. Bile acids were extracted from whole small intestine with its content. n = 3. B: Gallbladder bile acids. n = 3. C: Deconjugation of bile acids in fecal slurry in vitro. n = 4–5. All results are expressed as mean ± SEM.

Analysis of gallbladder and small intestine bile acid composition revealed that Gly-β-MCA was neither “gut restricted” nor resistant to gut bacteria-mediated deconjugation. To better characterize Gly-β-MCA metabolism in vivo, we next orally administered a single bolus of Gly-β-MCA (0.5 mg, which is about ∼10% of total daily intake at 160 mg/kg/day dose) to *Cyp2c70* KO mice and measured the appearance of Gly-β-MCA, deconjugated MCAs, and T-MCAs in small intestine and gallbladder bile. We found that 1 h after Gly-β-MCA administration, Gly-β-MCA and β-MCA appeared in the small intestine and gradually decreased at 4 h and 16 h (Fig. 6A). Since the small intestine transit time is less than 2 h and the total gastrointestinal transit time in mice is about 6 h (25, 26), these results confirmed that the administered Gly-β-MCA was deconjugated in small intestine to a significant extent before it reached the large intestine. In addition, T-β-MCA was detectable at 1 h and further increased significantly at 4 h and 16 h, suggesting that Gly-β-MCA-derived β-MCA was absorbed and transported to liver to be re-conjugated to T-β-MCA, which was released into small intestine. In contrast, α-MCA was not detectable in small intestine (Fig. 6A), suggesting that conversion of β-MCA to α-MCA did not occur at a measurable level in the small intestine over the 16-h period in vivo. At 16 h, ∼280 μg of T-β-MCA was detected in small intestine of Gly-β-MCA-treated mice, suggesting that ∼50% or more of the administered Gly-β-MCA entered the enterohepatic circulation after microbiota-mediated deconjugation (Fig. 6A). Consistent with this model, gallbladder T-β-MCA gradually increased to the highest level (∼20 μg) at 4 h and did not further increase at 16 h post Gly-β-MCA administration (Fig. 6B). Because mice were under fasting over the 16-h period, it was likely that the gallbladder was maximally filled, and T-β-MCA was continuously released into the small intestine instead of being continuously accumulated in the gallbladder. Gly-β-MCA was detected at the highest level (∼5 μg) in gallbladder at 4 h post Gly-β-MCA administration (Fig. 6B), supporting the model that Gly-β-MCA is not “gut restricted” and can be absorbed in the small intestine. The gallbladder Gly-β-MCA was not likely derived from β-MCA absorbed in the gut because mouse livers almost exclusively conjugate bile acids to taurine. At 1 h and 4 h post Gly-β-MCA administration, the gallbladder T-β-MCA level was ∼3–3.5 folds higher than Gly-β-MCA (Fig. 6B), suggesting that ∼75% of the administered Gly-β-MCA was absorbed in the form of β-MCA in the gut and re-conjugated to taurine in the liver. In an in vitro bile acid deconjugation assay using fresh fecal slurry from *Cyp2c70* KO mice, we found that ∼60% of T-CDCA-d4 was deconjugated, ∼7% of Gly-β-MCA was deconjugated, while ∼90% of T-UDCA-d4 was deconjugation over a 6 h incubation (Fig. 6C). This result suggests that, at least under in vitro condition, Gly-β-MCA shows a much slower deconjugation rate than other bile acids. In contrast, Gly-β-MCA was more extensively deconjugated in vivo during gastrointestinal transit and entered the enterohepatic circulation primarily after bacteria-mediated deconjugation.

### Gly-β-MCA treatment promotes fecal bile acid excretion and decreases colon LCA exposure

**Fig. 7:**
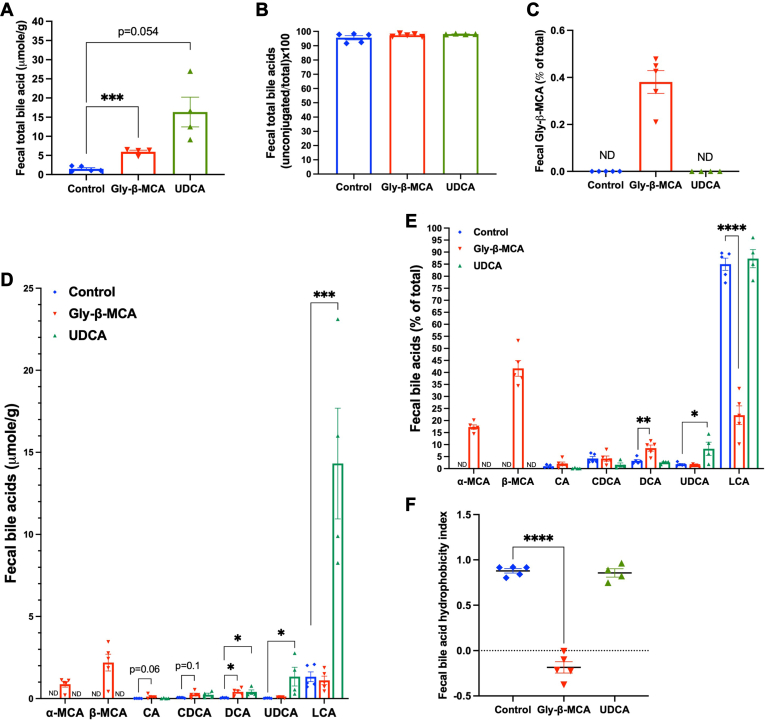
Gly-β-MCA and UDCA treatment effects on fecal bile acid composition in female *Cyp2c70* KO mice. Female *Cyp2c70* KO mice at 8 weeks of age were treated with Gly-β-MCA or UDCA (160 mg/kg/day) for 4 weeks and fecal samples from individual mouse were collected. A: Fecal total bile acids (normalized to fecal weight). B: Fecal unconjugated bile acids (% of total). C: Fecal Gly-β-MCA (% of total fecal bile acids). D: Fecal bile acid amount normalized to fecal sample weight (only unconjugated bile acids). E: Fecal bile acid composition (only unconjugated bile acids). F: Fecal bile acid hydrophobicity index. n = 4–5. All results are expressed as mean ± SEM. One-way ANOVA and Tukey post hoc test were used for all statistical analysis. A *P* < 0.05 was considered statistically significant. “∗”, <0.05; “∗∗”, <0.01; “∗∗∗”, <0.001; “∗∗∗∗” <0.0001.

To understand why neither Gly-β-MCA nor UDCA treatment expanded endogenous bile acid pool while Gly-β-MCA treatment paradoxically decreased endogenous bile acid pool by ∼25% in *Cyp2c70* KO mice (Fig. 4D), we next measured total fecal bile acids and found that Gly-β-MCA treatment increased total fecal bile acids by ∼4 folds, and UDCA treatment increased total fecal bile acids by ∼10 folds (Fig. 7A). Fecal bile acid composition, which closely reflects colon bile acid composition after microbiota-mediated biotransformation, was analyzed. Although mice treated with Gly-β-MCA or UDCA appeared to have reduced bile acid deconjugation in the small intestine (Fig. 5G), bile acid deconjugation was carried largely to completion in the large intestine in these mice (Fig. 7B). Gly-β-MCA accounted for only ∼0.4% of total fecal bile acids (Fig. 7C), which, about the Gly-β-MCA abundance in the small intestine (Fig. 5E), suggested that Gly-β-MCA deconjugation was essentially complete as it passed through the large intestine. Consistent with a previous report (14), the fecal bile acids of *Cyp2c70* KO mice consisted of ∼85% of LCA (Fig 7D, E), which was because T-CDCA and T-UDCA, which are substrates for LCA synthesis in the gut, together accounted for ∼80% of total gallbladder bile acids of *Cyp2c70* KO mice (Fig. 5A). Interestingly, Gly-β-MCA treatment not only increased total fecal MCA as expected, but also increased the endogenous bile acids CA (∼10 folds), CDCA (∼4 folds) and DCA (∼9 folds) compared to untreated controls (Fig. 7D). These results suggested that Gly-β-MCA treatment likely inhibited the absorption of the primary bile acids T-CDCA and T-CA in the small intestine since these bile acids were decreased in gallbladder bile and/or small intestine but increased in feces (Fig 5A, D). Decreased T-CA re-absorption in small intestine may also contribute to increased DCA synthesis in large intestine and excretion in feces (Fig. 7D). Overall, MCA accounted for ∼60% of total fecal bile acids in Gly-β-MCA-treated mice, which decreased relative LCA abundance to ∼22% from ∼85% in the untreated controls (Fig. 7E). Decreased hydrophobic LCA and increased MCA abundance resulted in significantly reduced fecal bile acid hydrophobicity (Fig. 7F). It should be noted that ω-MCA, which can be produced from β-MCA in the gut, was not measured in fecal samples due to technical limitation of cleanly separating ω-MCA from α-MCA and β-MCA by the LC-MS method. Otherwise, the relative abundance of fecal LCA and fecal bile acid hydrophobicity are likely lower should ω-MCA be incorporated into the calculation. In contrast, the markedly increased total fecal bile acid amount in UDCA-treated mice was largely driven by increased fecal LCA enrichment but to much less extent by fecal UDCA enrichment (Fig 7A, D), which reflected efficient UDCA to LCA conversion in the large intestine. As a result, LCA remained the major bile acid in the fecal samples of UDCA-treated mice, and UDCA treatment did not alter fecal bile acid hydrophobicity (Fig 7E, F). In summary, these results showed that Gly-β-MCA treatment increased fecal bile acid excretion of both endogenous bile acids and Gly-β-MCA-derived MCAs, which may contribute to decreased endogenous bile acid pool in mice. In addition, Gly-β-MCA treatment also caused large intestine to be exposed to significantly less hydrophobic bile acids. In contrast, UDCA treatment markedly increased large intestine flux of bile acids highly enriched with hydrophobic LCA.

## Discussion

A major finding of our study is that Gly-β-MCA is highly effective in reducing hydrophobic bile acid hepatobiliary toxicity and therefore has a potential clinical application in treating human cholestasis. Given to female *Cyp2c70* KO mice at a dose that was equivalent to the optimal clinical UDCA dose used in patients with PBC, Gly-β-MCA appeared to be as effective as UDCA in reducing serum liver enzyme, portal inflammatory infiltration, and ductular reaction and significantly more effective in attenuating portal fibrosis than UDCA. In cholestasis of various etiology, decreasing bile acid hepatobiliary toxicity is the major therapeutic goal to slow disease progression. This may be pharmacologically addressed by decreasing bile acid pool hydrophobicity and promoting biliary secretion (i.e., UDCA) or by decreasing enterohepatic bile acid pool (i.e., inhibition of bile acid synthesis by OCA or FGF19 analog or intestine bile acid absorption by apical sodium-dependent bile acid transporter (ASBT) inhibitors). Our study suggests that the therapeutic benefits of Gly-β-MCA may be attributed to its ability to simultaneously reduce the enterohepatic bile acid pool size and hydrophobicity. These findings also suggest that Gly-β-MCA treatment is beneficial in alleviating bile acid hepatobiliary toxicity in both male and female *Cyp2c70* KO mice, while the relatively modest improvement previously observed in male *Cyp2c70* KO mice treated with 20 mg/kg/day Gly-β-MCA was likely due to the low dose of Gly-β-MCA used.

By using *Cyp2c70* KO mice that do not synthesize endogenous MCAs, we found that orally administered Gly-β-MCA mainly followed a unique microbiota-dependent enterohepatic circulation to cause favorable changes in bile acid metabolism. Our findings showed that Gly-β-MCA could be absorbed either in its unmodified form or after being deconjugated to β-MCA. Direct Gly-β-MCA absorption was likely at a low rate compared to other endogenous conjugated bile acids that are known to be efficiently recycled in the ileum via ASBT. This was supported by previous reports that, when given orally at loose doses (10–20 mg/kg/day), Gly-β-MCA was barely detectable in gallbladder bile (17, 18). Following a single bolus of Gly-β-MCA administration, we estimated that ∼75% of the absorbed Gly-β-MCA entered the enterohepatic circulation following microbiota-mediated deconjugation. It was also estimated that more than 50% of the orally administered Gly-β-MCA may be absorbed based on the measured amount of gallbladder and intestine T-β-MCA following a single bolus of oral Gly-β-MCA administration, because the most plausible source of the T-β-MCA in *Cyp2c70* KO mice was gut-derived β-MCA. There results suggest that Gly-β-MCA does not act in a “gut restricted” manner, and by enriching the enterohepatic bile acid pool with MCA species, it significantly decreases the bile acid pool hydrophobicity and toxicity.

It was noted that neither Gly-β-MCA nor UDCA treatment expanded the endogenous bile acid pool, which may be explained by markedly increased fecal bile acid excretion in the treated mice. This reflects the tightly regulated bile acid homeostasis whereby increased fecal excretion fully compensates for the increased bile acid intake. It was also interesting to note that Gly-β-MCA paradoxically decreased the total bile acid pool by ∼25% despite a ∼30% enrichment of the endogenous bile acid pool with MCAs and induction of hepatic bile acid synthesis gene expression. Our data suggests that Gly-β-MCA is absorbed at a relatively lower efficiency and its presence may competitively inhibit intestine uptake of other endogenous bile acids in the ileum, which contributes to reduced enterohepatic bile acid pool. This was supported by evidence of reduced CDCA and CA abundance in biliary bile and small intestine and increased CDCA, CA, and DCA in the feces of Gly-β-MCA-treated mice. Our results also suggest that Gly-β-MCA treatment may be advantageous over UDCA in that Gly-β-MCA treatment caused the large intestine to be exposed to an MCA-enriched hydrophilic bile acid pool with markedly decreased relative abundance of the cytotoxic LCA. In contrast, the large intestine of UDCA-treated mice appeared to be exposed predominantly to UDCA-derived LCA, but not UDCA itself. The untreated *Cyp2c70* KO mice already exhibited a fecal bile acid composition containing predominantly LCA due to increased CDCA availability. UDCA treatment did not further increase fecal LCA abundance, but UDCA treatment caused a ∼10 folds increase of fecal bile acid excretion, suggesting a significantly increased LCA flux through the large intestine. It should be noted that although the enterohepatic bile acid composition resembles that of humans, the fecal bile acid composition of *Cyp2c70* KO mice, which closely reflects colon bile acid composition, is quite different from that of humans. This is because in humans, CDCA and DCA are major fecal bile acids with a lower abundance of LCA. In humans, UDCA treatment resulted in LCA being the major fecal bile acid (27). In contrast, LCA is already the predominant fecal bile acid in *Cyp2c70* KO mice, and therefore, UDCA treatment did not further alter the fecal bile acid composition in *Cyp2c70* KO mice.

Gly-β-MCA was initially developed as an “gut-restricted” FXR antagonist (17). However, while intestinal FXR antagonism by Gly-β-MCA was shown to be beneficial in improving metabolic health in obesity, it may be an undesirable effect of cholestasis treatment given the protective role of FXR against intracellular bile acid accumulation (9). In humans, *FXR* loss-of-function mutations caused progressive familial intrahepatic cholestasis type 5 (PFIC-5) with undetectable hepatic BSEP expression and intrahepatic bile acid accumulation (28, 29, 30). Gly-β-MCA treatment indeed increased hepatic CYP7A1, CYP8B1 and NTCP mRNA expression, suggesting attenuated hepatic FXR signaling. However, FXR antagonism by Gly-β-MCA treatment was likely inconsequential because Gly-β-MCA treatment increased hepatic BSEP expression and decreased hepatic bile acid accumulation in *Cyp2c70* KO mice. Collectively, our data suggest that the anti-cholestasis effect of Gly-β-MCA may be mainly dependent on its unique physicochemical and pharmacokinetic properties independent of its FXR antagonism function.

The use of *Cyp2c70* KO mice without endogenous MCA synthesis was critical in identifying the anti-cholestasis effect, pharmacokinetics, and mechanisms of action of Gly-β-MCA, which are expected to be largely masked in mice with endogenous MCA synthesis. However, we also note that Gly-β-MCA treatment in *Cyp2c70* KO mice should not be considered restoring MCA loss to re-establish murine bile acid pool composition because the kinetics and functional properties of Gly-β-MCA and endogenously synthesized T-MCAs are very different and not interchangeable. Murine bile contains only trace amount of T-CDCA, while, similar to humans, *Cyp2c70* KO mice have abundant biliary T-CDCA. Gly-β-MCA treatment did not eliminate the presence of T-CDCA in *Cyp2c70* KO mice. Therefore, the bile acid pool composition of Gly-β-MCA-treated *Cyp2c70* KO mice does not resemble that of WT mice, but rather informs what the human bile acid pool composition may be after Gly-β-MCA treatment. Future studies may test the potential anti-cholestasis effect of taurine-β-MCA and β-MCA in *Cyp2c70* KO mice. These hydrophilic bile acids are expected to enrich the endogenous bile acid pool and reduce bile acid pool hydrophobicity. However, unlikely Gly-β-MCA, these bile acids may be efficiently absorbed in the ileum, and their impact on endogenous bile acid absorption, overall bile acid pool size, and bile acid-microbiome interaction in the large intestine remain to be determined.

In summary, evidence provided in this pre-clinical study demonstrates that Gly-β-MCA is a potent anti-cholestasis agent. Gly-β-MCA is a hydrophilic and non-cytotoxic bile acid and likely has a favorable safety profile. Gly-β-MCA may hold the potential as a therapeutic agent for treating human cholestasis.

## Data availability

All data described in the manuscript are contained within the manuscript.

## Conflict of interest

The authors declare that they have no conflicts of interest with the contents of this article.
